# Transition of adolescents from paediatric to adult HIV care in South Africa: A policy review

**DOI:** 10.4102/sajhivmed.v26i1.1674

**Published:** 2025-04-02

**Authors:** Charné Petinger, Talitha Crowley, Brian van Wyk

**Affiliations:** 1 School of Public Health, Faculty of Community and Health Sciences, University of the Western Cape, Cape Town, South Africa; 2 School of Nursing, Faculty of Community and Health Sciences, University of the Western Cape, Cape Town, South Africa

**Keywords:** policy review, adolescents living with HIV, transfer of care, transition, HIV, guidelines, adolescents, South Africa

## Abstract

The successful roll-out and improved delivery of antiretroviral therapy (ART) services has led to paediatric HIV patients surviving to reach adolescence. Adolescents living with HIV (ALHIV) are challenged when transitioning to adult HIV care programmes where they must negotiate new care pathways, changes in healthcare providers and self-manage their chronic condition, in addition to dealing with the psychological and physiological developmental changes of adolescence. The transition process needs to be well guided, to ensure that ALHIV on ART maintain optimal adherence and remain engaged in care. Viral suppression and retention in care are significantly lower for older adolescents (15–19 years) compared to children and younger adolescents under 15 years – coinciding with the post-transition period. Comprehensive and structured transition protocols may have a significant impact on positive health outcomes. In sub-Saharan Africa, there is a dearth of policies and implementation guidelines for ALHIV who are transitioning to adult HIV care. The current review reports on policies and guidelines for transitioning ALHIV to adult HIV care in South Africa. Eight policies were identified, which were developed at global (*n* = 2), national (*n* = 2) and provincial levels (*n* = 1), and guided implementation (*n* = 3). Current national and provincial policies provide guidance on when to transition a patient clinically to facilitate the switch to adult ART regimens. Although global policies and implementation guidelines emphasise specific and comprehensive care for ALHIV on ART, these are not carried over to national and provincial policies in South Africa. Further development of policies is required to guide comprehensive, adolescent-friendly transition processes for ALHIV on ART in South Africa.

**What this study adds:** This policy review highlights the gap in national health policies and guidelines to guide the transition process of adolescents from paediatric to adult HIV care.

## Introduction

The rapid development and improved delivery of antiretroviral therapy (ART) services contributed to children living longer with HIV and now reaching adolescence.^1^ Adolescents (aged 10–19 years) are the fastest-growing population group with HIV because of both improved survival and a high HIV infection rate.^2^ Adolescents living with HIV (ALHIV) are recognised as a ‘priority population group’.^3^ The adolescent period is characterised by prominent physical, cognitive, and emotional developmental changes in addition to social barriers that challenge persistent adherence to ART and engagement in care.^1,4^ It is noted that older adolescents (aged 15–19 years) report significantly lower viral suppression rates than younger adolescents (aged 10–14 years); which indicates problems with adherence and retention in care after transition to adult HIV care programmes.^5^

It is estimated that approximately 320 000 ALHIV will transition from paediatric to adult HIV care in South Africa by 2028.^6,7^ In South Africa, ALHIV are also expected to transfer from paediatric to adult HIV care around the ages of 10–13 years.^8,9^ This transfer to adult care is done to facilitate better clinical outcomes, such as adherence, viral load suppression, and reducing the development of comorbidities and long-term drug-related toxicities. In addition, the regimen changes to a more simple, fixed-dose combination.^10,11^ Paediatric HIV paediatric care is not commonly associated with fixed-dose combinations – in spite of National ART Clinical Guidelines – and this switch mostly often occurs upon transfer to adult HIV care.^10,12^ Adult HIV programmes are described as being fairly insular, differing from paediatric settings where the care is comprised of an interdisciplinary team, and where the patient has a closer relationship with the healthcare providers.^4^ It is essential that the transition process to adult care programmes be purposeful and planned to ensure that ALHIV transition successfully into adult HIV care and remain adherent and engaged in care.^2^ The key elements to a successful transition involve preventative measures, which include ensuring that ALHIV are ready to transition, have access to care that does not affect their education, have a support system both at home and at the healthcare service providers, are knowledgeable about HIV and the transition process, and have access to psychosocial support to address individual challenges.^2,13^

Managing transition becomes particularly challenging in sub-Saharan African countries marked by high HIV prevalence and resource constraints, where healthcare systems are not adapted to the specific needs of ALHIV.^2^ Even though South Africa is a middle-income country, it is characterised by huge discrepancies between low- and high-resourced settings.^14^ It is reported that ALHIV in South Africa are often underserved because of limited adolescent-specific HIV services, which results in poorer access and adherence to ART.^15^ In addition, ALHIV face the challenges of HIV-related stigma, a lack of organised systems within health services that impede ART access, and lack of psychosocial support.^16^

A review of the transition of care of ALHIV in sub-Saharan African countries indicated several challenges, while noting very limited data on outcomes and guidelines.^2^ While there are several supportive guidance protocols in place for transition in high-income countries, these may not be readily applicable to the South African context.^14^ In South Africa, age (reaching 12–13 years) of ALHIV is used as a guide for health workers when deciding when transition should take place. However, Zanoni et al. found that the age of transitioning was not associated with viral suppression after undergoing the transition to adult HIV care.^17^

### Problem statement

A systematic review of transition interventions found that transition practices that prioritise communication from and between healthcare workers, psychological and social support, group transition, mHealth, education, and individualising care plans produce more effective health outcomes for ALHIV after transitioning to adult care.^13^ Further research found that adequate transition support increased adolescents’ knowledge and ability to manage their condition, which increased the likelihood of adherence, retention in care and viral load suppression.^9,18^ Zanoni et al.^17^ developed a tool to assess readiness for transition among ALHIV, and concluded that transition is not a guided process but rather informed by the age of the ALHIV.

It is argued that structured, comprehensive transition protocols for ALHIV are imperative to reach the Joint United Nations Programme on HIV/AIDS (UNAIDS) goals for treatment uptake and success in this priority population.^15^ This is even more pronounced in the context of overburdened and poorly functioning health systems in the African context, where the brunt of the HIV pandemic amongst adolescents is situated. It is therefore necessary to review current health policy and regulations in South Africa to identify how transition from paediatric to adult HIV care should take place, and how these policies align with global recommendations. This will be done through the identification and review of national policies related to the transition or transfer of ALHIV to adult HIV care. Furthermore, a policy review will highlight gaps in policy and regulation, and inform the subsequent development of evidence-based policy and health systems practice towards improved transition of ALHIV from paediatric to adult HIV care in the South African and other contexts with hyper-HIV epidemics.

### Objective

To review current policies that guide the transition from paediatric to adult HIV care for ALHIV in the public sector services in South Africa.

## Research methods and design

### Policy review

This policy review seeks to describe the current policies underpinning the transition process for ALHIV transitioning from paediatric to adult HIV care in South Africa. Through the examination of policies, this review allows us to assess and identify the ways in which the policy and guideline address the requirements.^19,20^ Within the context of this review, we can assess whether the included policies and guidelines address the transition or transfer to adult HIV care for ALHIV. The health policy analysis (HPA) triangle developed by Walt and Gilson^21^ was applied to identify policy context, content, process, and actors.^22^ The framework provides a comprehensive understanding of the policy process as well as a way to examine its nature, which is done through an examination of the process in which a policy is formulated, designed, implemented, and evaluated.^23^ It applies a new paradigm of thinking wherein all factors that may affect the implementation of health policies are considered for a wide range of health issues.^24,25^ It can be applicable for the examination of policies that inform the transition from paediatric to adult HIV care in a South African context. The framework was therefore used as a guide for the effective identification of particular components of the selected policies and guidelines.

### Data collection

To identify suitable policy documents, the inclusion criteria were as follows:

- the policy document must include ALHIV;
- the document must apply to the South African context; and
- the document must include information on the transition or transfer from paediatric to adult HIV care.

The internet and the South African Knowledge Hub were used to search for policies related to the topic published in the last 10 years (2014–2024) in the English language. The Knowledge Hub is an E-library of the South African Government that provides access to resources such as policy documents. The included search terms were ‘HIV’, ‘Adolescents’, and ‘Transition in care’. The search string for the Knowledge Hub was ‘Adolescent HIV’. In addition, documents obtained from experts in the field of adolescent HIV care were added.

### Data analysis

Content analysis was applied to examine the meanings, patterns or themes that emerged in the document text.^26^ The steps followed in the content analysis included preparing the text, immersion in the data, making notes of the information in the text, defining units of analysis using themes, organising the data comprehensively through coding, coding all of the text, drawing conclusions from the coded data, and finally, describing and interpreting the results.^27^ This method was useful in this review as it allows the description of documents through examining what is being said and by whom, and to whom it is directed.^28^

### Ethical considerations

Ethical clearance to conduct this study was obtained from the University of Western Cape, Biomedical Science Research Ethics Committee (reference no. BM23/6/5). The article followed all ethical standards for research without direct contact with human or animal subjects.

## Results

### Description of selected policies

Eight policies met the inclusion criteria and were included for analysis. The summary characteristics of the policies and guidelines are described in Table 1. Using Walt and Gilson’s policy analysis framework,^21^ we describe the context, content and actors (policy developers). The documents were described as to whether it is a global-level, national, provincial, or implementation-level policy. Policies, guidelines, and toolkits from the national level to the implementation level are included within this analysis to describe the current guidance that is available for ALHIV who are transitioning from paediatric to adult HIV care. A policy can be understood as a comprehensive systematic plan, programme and strategy containing principles and values to guide and inform decision-making and practice.^29^ It refers to formal guidelines for how governmental decisions should be made and how programmes should be implemented.^29^ Moreover, policies could also be used to inform legislation and regulations.^30^ Guidelines, however, refer to the general policy principles, rules, or advice that may be used to promote action.^29^ Toolkits refer to certain sets of recommendations whereby insight is gained from research and can be used to inform policymakers.^31^

**TABLE 1:** Summary characteristics of included policies (*N* = 8).

Context	Policy title	Year	Primary content	Content related to ALHIV and transitioning	Policy developers
Global level	Consolidated guidelines on HIV prevention, testing, treatment, service delivery and monitoring^32^	2021	Providing guidance on HIV testing, prevention, treatment, and care.	Transitioning involves additional challenges such as increased responsibility for their own care, disclosure issues, difficulties navigating the healthcare system, lack of links between adult and paediatric services, and inadequately skilled healthcare providers.	WHO
	HIV/TB clinical guide for primary care^33^	2018	Practical guidelines for clinicians working with patients living with HIV in primary healthcare settings.	Global epidemiology of adolescents, unique challenges of ALHIV, key recommendations to address the challenges, and general recommendations.	MSF
National level	2023 ART clinical guidelines for the management of HIV in adults, pregnancy and breastfeeding, adolescents, children, infants and neonates^12^	2023	A reference guide for ART in adults, pregnant and breastfeeding women, adolescents, paediatric clients for healthcare workers. Focusing on providing guidance on initiation of ART, returning to care, switching clients to dolutegravir-containing regimens, routine management of ART to promote viral suppression, and highlighting critical areas of integrated ART, TB and family planning services, and the use of differentiated models of care.	Minimal information on when and how to clinically transition to adult ART regimens.	South African National Department of Health; WHO
	2023–2028 National Strategic Plan for HIV | TB | STIs^34^	2023	The goals of the NSP are to break down barriers to achieving solutions for HIV, TB and STIs; maximise equitable and equal access to services and solutions for HIV, TB and STIs; fully resource and sustain an efficient NSP led by revitalised, inclusive, and accountable institutions; and to build resilient systems for HIV, TB and STIs that are integrated into systems for health, social protection, and pandemic response.	Approaches, interventions, or services that can be provided to the population to maximise equitable and equal access to services. Ensuring that practices are adolescent-friendly, and to prepare and plan for transition to adult care.	South African National AIDS Council; National Department of Health; The Global Fund to Fight AIDS, TB, and Malaria; UNAIDS; WHO; UNICEF
Provincial level	The Western Cape consolidated guidelines for HIV treatment: prevention of mother-to-child transmission of HIV (PMTCT), children, adolescents and adults^35^	2020	A combination of guidance on HIV treatment across the life course and care continuum.	Providing guidance on when and how to transfer patients on paediatric regimens to adult ART regimens.	The Western Cape Department of Health, Adult and Paediatric HAST (HIV, AIDS, STI and TB) policy advisory groups; Medicines Information Centre University of Cape Town
Implementation level	Guidelines for adherence to antiretroviral therapy in adolescents and young adults^36^	2017	Providing guidance and support to maintain ART adherence for adolescents: the individual, family, community and cultural structures, health services, and medication.	Providing practical guidelines to support and improve adherence and facilitating a smooth transition.	WHO; Southern African HIV Clinicians Society
	Adolescent and youth transition of care toolkit^37^	2020	Guidance for healthcare providers and multidisciplinary teams of children, youth, and adolescents to implement and monitor the transition from paediatric to adult care.	Different forms of transitioning to adult care, outlining steps to achieve successful transition. Barriers to transition: structural, facility, family, peer, and individual levels.	The New Horizons Collaborative; The Elizabeth Glaser Paediatric AIDS Foundation; Johnson & Johnson
	Working with adolescents living with HIV: A toolkit for healthcare providers^38^	2016	Developing skills and competencies for healthcare workers rendering care for ALHIV that is suitable to their needs.	Steps that should be considered throughout the transitioning process, highlighting the importance of supportive care geared towards adolescents and their specific needs.	USAID; PEPFAR; Wits RHI; Southern African HIV Clinicians Society; Gauteng Department of Health; North-West Department of Health

Note: Please see the full reference list of this article, Petinger C, Crowley, T, Van Wyk, B. Transition of adolescents from paediatric to adult HIV care in South Africa: A policy review. S Afr J HIV Med. 2025;26(1), a1674. https://doi.org/10.4102/sajhivmed.v26i1.1674, for more information.

ALHIV, Adolescents living with HIV; ART, antiretroviral therapy; MSF, Médecins Sans Frontières [Doctors without borders]; NSP, National Strategic Plan; PEPFAR, United States President’s Emergency Plan for AIDS Relief; PMTCT, prevention of mother-to-child transmission; RHI, Reproductive Health and HIV Institute; STI, sexually transmitted infection; TB, tuberculosis; UNAIDS, Joint United Nations Programme on HIV/AIDS; UNICEF, United Nations Children’s Fund; USAID, United States Agency for International Development; Wits, University of the Witwatersrand.

The first document is the WHO’s consolidated guidelines on HIV prevention, testing, treatment, service delivery and monitoring.^32^ These guidelines are also used to inform the South African national ART guidelines. It does not primarily focus on transitioning but provides guidelines and recommendations to ensure seamless transitioning to adult HIV care to improve health outcomes for adolescents.

The second is the HIV/TB (tuberculosis) Clinical Guide for Primary Care,^33^ which is geared toward being a practical guidance tool for healthcare workers to provide comprehensive care relating to HIV and TB, in primary care settings. This guideline does not specifically focus on adolescents as the primary population, but rather on all people with HIV.

The third is the South African National ART Clinical Guidelines for the management of HIV in adults, pregnancy and breastfeeding, adolescents, children, infants, and neonates.^12^ This document provides clinical guidance on when adolescents are to be transferred to adult ART regimens.

The fourth is the National Strategic Plan for HIV, TB, and STIs (sexually transmitted infections) 2023–2028.^34^ The document centres around prevention, care and management of HIV, TB, and STIs, and enhancing access to services.

The fifth is the Western Cape consolidated guidelines for HIV treatment: prevention of mother-to-child transmission (PMTCT), children, adolescents, and adults.^35^ Similarly, the guidelines do not specifically focus on adolescents transitioning to adult care but provide clinical guidelines on when to transition adolescents.

The sixth is the Southern African HIV Clinicians Society Guideline: Adherence to Antiretroviral Therapy in Adolescents and Young Adults, which provides a more in-depth discussion of the needs of adolescents, barriers to transitioning, and key guidelines to ensure optimal transitioning.^36^

The seventh is the New Horizons adolescent and youth transition of care toolkit, which is the only policy document found that solely guides the transition processes for adolescents.^37^

The final policy is the Wits Reproductive Health and HIV Institute guideline: *Working with ALHIV: A toolkit for healthcare providers*.^38^ This guideline discusses the clinical management of HIV for ALHIV and the psychosocial and mental well-being of ALHIV.

## Emerging themes

Eight policy documents were included in the final analysis. The documents addressed the transition from paediatric to adult HIV care in two primary ways (*clinical guidelines* and *transition as a psychosocial process*). The findings discuss the key information which stood out from the policies, and evaluate how the content of the policy documents provides guidance on the transition.

### Clinical guidelines

Two documents addressed the transition from a clinical standpoint only, discussing the change in regimen that adolescents must go through, which is understood as the transition, from the age of 10 years old, in order to assist with adherence as they change to a fixed-dose combination regimen.^12,35^

### Transition as a psychosocial process

Six of the eight selected documents acknowledged the associated factors that accompany the transition process.^32,33,34,36,37,38^ These documents highlight the importance of providing comprehensive care in the form of adolescent-friendly services that are suited for adolescents and the unique challenges they face. Adolescent-friendly services are geared towards ensuring that adolescents obtain the healthcare they require by overcoming unique barriers, through non-judgemental and confidential care, appealing to adolescents, and ensuring that they are aware of the services offered to them.^39^ The following points discuss the common guiding principles that stood out from the abovementioned eight documents:

#### The transition must be collaborative

It is recommended that before and during transitioning, healthcare workers from the paediatric services and adult services should be linked and should communicate about the adolescent’s care management.^32,37,38^ Moreover, planning and collaboration are also recommended between the adolescents, their caregivers, and healthcare providers to ensure a mutual understanding concerning what is required and what will change. This is an important aspect to consider, as it is apparent that the clinical guidelines recommend that adolescents be transferred to adult regimens as early as 10 years old, should they meet the requirements.^12,35^

#### Adolescents should be adequately prepared

Transition readiness is important, as it is found that when adolescents are adequately prepared to transition to adult services, their health outcomes, such as adherence and retention in care, are positive.^9^ This includes ALHIV knowing about HIV and their care management, being fully disclosed to about their HIV status, being familiar with how the adult services would be different or similar, knowing how to access services, collaborating with healthcare providers and their caregivers on managing their treatment plans, and establishing their own goals related to their care.^32,33,36,37,38^

#### Healthcare workers should be competent in providing care to adolescents living with HIV

It is recognised that adolescents have challenges and needs that are unique,^32^ and therefore require adolescent-friendly service delivery. However, the documents, particularly the South African-specific clinical-based guidelines, do not unpack the components of adolescent-friendly services and how they should be implemented.^12,34,35^ It is, however, noted that healthcare services should provide peer support or adherence groups, wherein adolescents can share and receive information about the experiences of people their own age.^33,37^ Moreover, confidentiality and disclosure issues should be heavily considered by service providers.^33,37^ Healthcare workers, specifically HIV care teams, should be trained in providing care for adolescents.^32,33,36^

### Evaluating policy content

The transition process involves ALHIV taking charge of their own health and disease management, which raises challenges for their engagement in care.^2^ A successful transition improves the health outcomes and treatment skills among adolescents.^40^ It is therefore necessary to evaluate whether existing transition practices and guidelines address the specific needs of adolescents and are optimised to their requirements and available resources, particularly within a low- or middle-income country context.^41,42^ The policies, guidelines and toolkits included in this review represent the currently available guidance on transition within the South African context. However, it is necessary to ascertain whether these documents are geared towards ensuring a successful transition from paediatric to adult HIV care for ALHIV. The policy analysis framework, as discussed previously, by Walt and Gilson,^21^ can also be used to inform whether these documents address the needs and unique challenges of ALHIV who are expected to transition from paediatric to adult HIV care.

All of the policy documents discuss the transition, albeit differently. We identified only one document where the transition process is the primary focus.^37^ This emphasises the need for policies that prioritise the transition to adult HIV care in its entirety. Furthermore, a precise definition of the transition is lacking in policy documents such as the National ART Guidelines and Western Cape Consolidated Guidelines,^12,35^ which only discuss the transition as a change in clinical regimens. In terms of context, there are four documents that were developed within the South African context;^12,34,35,36^ however, the implementation status of some, such as the National Strategic Plan (NSP) and Southern African HIV Clinicians Society guidelines, are unclear.^34,36^ It is also evident that only four documents provide guidance on how to transition adolescents effectively by providing recommendations on overcoming adolescent-specific barriers.^32,33,36,37^

## Discussion

It is evident that the guidelines discuss specific care that should be provided to ALHIV. However, the NSP^34^ is the only governmental document that acknowledges the transition process as a whole, whereas the clinical guidelines prioritise the change in regimens.^12,35^ The implementation of the NSP, as well as the other implementation-level guidelines,^36,37,38^ is unclear. Within the higher-level policies, such as the NSP,^34^ they provide a framework and guiding principles about the services and care that should be provided to ALHIV. However, guidelines such as the Western Cape Consolidated Guidelines and National ART Clinical Guidelines are more medically focused, with an absence of guidance of services and care specifically geared toward the entire transition to adult HIV services for ALHIV.^12,35^ This is strengthened by a recent review finding that there are limited transition policies and protocols at both the national and clinical levels.^13^ The development of national-level policies tailored to the unique experiences and needs of ALHIV who are transitioning from paediatric to adult HIV care is necessary, as the current practices within South Africa are found to result in poor engagement in care and inconsistent timing of the transition.^7^ Optimising policies and practices would ultimately lead to successful health outcomes for ALHIV.^1,7,43^ The lack of policies addressing the experiences and needs of ALHIV, during this adolescent period specifically, highlights the lack of recognition being brought upon care that is provided to adolescents, showcasing an increased focus on paediatric and adult care, separately.

Although the implementation-level guidelines bring forth practical guidelines on providing care for adolescents, it is unclear whether these are implemented.^36,37,38^ Mark et al.^44^ found that there remain major gaps in turning policy into practice. Moreover, the global-level policies that are discussed throughout this article provide adolescent-friendly guidance toward the transition, although the suitability of implementing it within the South African context is uncertain.^32,33^ Thus, there is a need for developing policies that are tailored and applicable to the South African context.^13^ Furthermore, these policies should encompass a clear definition of transition, the age of transition, and the required facets which would ensure a successful and effective transition.^44^

All documents attempt to describe and emphasise positive health outcomes and ensure adherence to HIV care for ALHIV. However, there should be guidelines or policies in place which not only prioritise the clinical-based regimen change for adolescents but also the accompanying, adolescent-specific factors which are implicated within the transition process. This includes psychosocial support, access to care, readiness to transition, and effective communication between different healthcare providers as well as caregivers. Moreover, applying a theoretical framework such as the biopsychosocial model to develop policies would be beneficial to ensure they are inclusive of adolescent-specific factors. Okonji et al.^45^ explain that using the biopsychosocial model is effective in developing interventions to improve ART adherence and retention in care. This is because it acknowledges that HIV management, especially for ALHIV, surpasses the biomedical process, and that the accompanying psychological and social processes have an influence on the management of HIV care.^45,46^ This should also consider the overburdened context of the public healthcare system in South Africa. It can be mitigated through effectively identifying ways in which adolescent-specific and supportive care without adding to the current constraints of staff shortages, untrained staff, and austerity measures.^44^

Therefore, it is evident that developing policies and guidelines that include the effects social, structural, and psychological processes have on disease management and treatment adherence may be beneficial to improving health outcomes.

## Conclusion

This policy analysis describes the current policies guiding the transition from paediatric to adult HIV care for ALHIV in South Africa. It is evident that current national and provincial policies^12,35^ do not provide guidance on providing comprehensive care for the transition process for ALHIV on ART. The national and provincial policies are primarily driven by the benefits of the clinical regimen change, but with little regard for the psychosocial processes and adaptations that are essential for successful post-transition engagement in care and maintaining optimal adherence and viral suppression. Whereas existing implementation guidelines provide valuable direction for health service delivery to ALHIV, these are not regulated and are therefore not consistently applied at primary healthcare facilities. Thus, we emphasise the need for policies to be developed that incorporate the unique challenges that adolescents face, as well as providing feasible guidance on providing services that are equitable and accessible.
